# Designing Immersive Sustainable Food Experiences in Augmented Reality: A Consumer Participatory Co-Creation Approach

**DOI:** 10.3390/foods11223646

**Published:** 2022-11-15

**Authors:** Dai-In Danny Han, Sílvia Gabriela Abreu e Silva, Kay Schröder, Frans Melissen, Mata Haggis-Burridge

**Affiliations:** 1 Academy for Hotel and Facility, Breda University of Applied Sciences, 4817 JS Breda, The Netherlands; 2 Research Centre Future of Food, Zuyd University of Applied Sciences, 6419 DJ Heerlen, The Netherlands; 3 Human Data Interaction Lab, Zuyd University of Applied Sciences, 6411 CR Heerlen, The Netherlands; 4 Academy for Games, and Media, Breda University of Applied Sciences, 4817 JT Breda, The Netherlands

**Keywords:** storytelling, immersive experience, sustainability, food, augmented reality

## Abstract

In light of the current debate on the impact of our current food system on climate change and related mitigation strategies, addressing the acceptance of sustainability aspects within consumer behavioral issues is of vital importance. However, the field remains mute on how those strategies can be designed and employed effectively to stimulate sustainable food consumption behavior. Immersive narrative design is a promising approach to engaging consumers in this context. Within this study, we shed light on how to create immersive, impactful, interactive narratives in augmented reality (AR) together with consumers. We propose a novel approach to how those stories can be planned, utilizing participatory design methods. Within a step-wise process, we develop the storyboard together with consumers. In the next step, we evaluate multiple approaches with AR application developers on how this storyline can be enhanced in AR considering the perspective of various stakeholders like developers, behavioral scientists, and consumers. Finally, we propose a conceptual framework for how immersive narratives can be designed and validated in a collaborative, multidimensional approach for impactful AR narrative content designs to stimulate sustainable food behavior for consumers.

## 1. Introduction

Sustainability has been a much-studied and debated topic in the food service and hospitality industries. Fueled by the announcement of the sustainable development goals in 2015, many organizations across industries have made attempts to embed sustainable practices and stimulate consumers to make more sustainable choices while engaging with their products and services [1]. Although organizations and people are increasingly aware of sustainability issues and the potentially detrimental effects of many of our current consumption behaviors on the environment and societies, it has remained a challenge to stimulate people to make more sustainable choices for their own well-being, the well-being of others, and the planet [2]. This has been particularly difficult in the food service and hospitality industries, which are often associated with enjoyment and affluent consumption patterns. The World Health Organization concluded that current food consumption patterns in developed countries are a key contributor to obesity issues and greenhouse gas emissions responsible for climate change [3]. Five evolutionary human tendencies were argued to make it difficult for us to behave sustainably despite our rational understanding of underlying issues [2], one of which includes the propensity to disregard impalpable concerns. In other words, we find it difficult to make rational decisions related to issues that seem too abstract and distant from our immediate personal lives.

Prior attempts were made to address this challenge and depicted immersive technologies and related information systems as promising avenues to stimulate more sustainable consumer behavior [4]. They argued that immersive technologies allow consumers to engage in experiences beyond our physical and geographical limitations, creating a higher sense of relatability, awareness, and an enhanced view of the world. Several studies have been conducted relating to addressing sustainability issues through immersive technologies. For instance, one study depicted the consequences of wasteful energy consumption through virtual environments [5], while another studied the effects of virtual environments to address environmental pollution through serious games [6]. They concluded that the higher perception of contingency led to greater self-efficacy and environmental behavior as well as increased support for environmental policy. A recent study concluded that immersive experiences in virtual reality that can evoke a sense of presence in the virtual environment could be an effective way to enhance empathy and positive behavioral intentions [7]. Augmented reality (AR) could be a promising tool to make abstract concepts such as sustainability tangible for consumers through the augmentation of digital content in the immediate environment to address our evolutionary tendency to be prone to disregard impalpable concerns [2]. Companies such as QReal have introduced lifelike 3D AR models to assist consumers in making food choices [8]. Current developments in immersive content in AR allow for interactive narrative content designs that are able to engage consumers through active interaction in the narrative [9]. AR offers opportunities to enhance sensory properties and create new food consumption experiences for consumers through its capability to create experiences in the first-person view which involve consumers as actors in the narrative. However, this requires a design of content that is highly applicable and relevant to the consumer’s personal context. Interactive digital narrative development is a field that has received more attention in recent years [10]. Although much can be learned from the film or games industries, a common approach for interactive narrative design is still undefined. Although a designer-driven approach is recognized as an efficient way to create content, it entails the risk of framing end-users into a narrative and disregards the possibility of reaching the maximum impact that could be possible through consumer-driven content designs. Participatory co-creative design processes with consumers are a recognized approach in service design in which designers involve consumers in the design process to create solutions that address the consumer’s pain point or objective accurately [11]. A similar approach to guided consumer participatory co-creation in the design of interactive content narratives for immersive narratives through AR is suggested in this paper to create highly relevant and convincing consumer content in the design process. Although such consumer-involving prototype tests are common practice in game design, to the best of our knowledge, it is unknown whether the involvement of consumers in the initial drafting of the narrative is an effective approach to interactive narrative content design for immersive technologies. As an increasing number of emerging consumer technologies is introduced to the market (e.g., AR, VR, smart technologies, service robots), this study aims to provide an evaluation of the effectiveness of design tools and propose a framework to facilitate participatory co-creative design processes with consumers to create impactful interactive content narratives for AR applications which could be key to address current sustainability-related consumer challenges. The narratives in the context of this study seek to stimulate consumers to make sustainable food choices through the use of mixed-reality headsets. Therefore, the following research question will be addressed: How can interactive content narratives in AR be effectively designed in a participatory co-creative design process with consumers for impactful narratives? In answering this research question, this study will offer an evaluation of design tools used for this purpose and discuss challenges and future opportunities for impactful consumer-driven co-creative AR interactive narrative design resulting in proposing a framework for participatory co-creative narrative design for immersive technologies that could maximize the impact on consumer attitudes to stimulate more sustainable behavior.

### 1.1. Consumer Co-Creation

Co-creation is the process of producing new value that is both material and symbolic which is cooperative, collaborative, concurrent, and peer-to-peer [12]. Consumers’ voices may be incorporated from a wide variety of stakeholder voices in the invention process through co-creation. Consumers may convey their ideas, talents, and capacities when they are involved in the product design and development process. As a result, the intention is to produce distinctive values created through co-creation that will benefit the organization [13]. Perspectives in the tourism context indicate that co-creation has evolved to become more about shared experiences than about potential financial gain [14]. In the business context, along with collecting innovative ideas, transferring information, and exchanging technology, co-creative partnerships attempt to cut costs and risks [15]. The digital environment allows for consumer participation in a co-creative process which was previously limited due to a lack of consumers’ expertise in product design and development [16].

To facilitate interactions between businesses and consumers, the Dialogue-Access-Reflexivity-Transparency (DART) model [17] provides an encompassing approach. *Dialogue* refers to cooperative learning and communication with stakeholders. *Access* is the process of obtaining knowledge about the experiences, facts, resources, abilities, and knowledge of others. The results of the process are highlighted through *reflexivity*. Visibility in the information flow is implied by the term *transparency* [17]. Other authors propose a two-dimensional model of the co-creation typology with customers to indicate their level of autonomy and involvement [18]. In the *X*-axis, *contribution activity* ranges from fixed to open, meaning the extent to which a company cedes control and engages its consumers as active players. The *Y*-axis, *selection activity* ranges from being mostly fixed by a company to being completely open to customer input, and the choice of these contributions can be made either by a company or by customers. The axis intersection generates four types of customer co-creation: submitting (fixed, firm-led), co-designing (fixed, customer-led), tinkering (open, firm-led), and collaborating (open, customer-led).

A greater number of consumers desire more control and authority over the creation of products and services. More autonomy can lead to increased levels of psychological ownership and intrinsic motivation, which in turn encourages creativity by making the creative work more pleasurable and gratifying [18]. This is a particularly relevant avenue to explore for hospitality, tourism, and leisure contexts, in which consumers are directly involved in the co-creation of their own experiences. Hence, this study aims to evaluate a design sprint developed to co-design stories (interactive content narratives) used to stimulate consumers to make sustainable food choices and identify challenges and future opportunities for the consumer-driven co-creative process.

### 1.2. Interactive Narrative Design

Interactive narrative design was defined as a compromise between narrative and interactive as two explicit design domains [19]. In their perspective, narrative design occurs on a holistic level, while interactive design deals with the granular, operational aspects of the design. It was revealed that narrative design included exclusive design parameters including characters, the environment, narrative themes, and a dramatic arc. The challenge lies in combining these parameters in a chain of events that is perceived by the audience as a rich story. Such narrative design outcomes often draw on emotional outcomes and are therefore designed using characters that the audience can identify and relate to [19]. In comparison, the successful use of interactive design can enhance the narrative through feelings of agency creating immersive experiences for the interactor [20].

The challenge lies in framing a common platform in which both aspects make sense together. To frame this platform, it was suggested to start with a fixed narrative which is overlayed with interactive elements through the development of characters within the narrative [21]. This approach is based on the video games context and thus leans on a development framework much related to how players interact with games [21]. For instance, continuous and freedom of interaction were recommended as key components to engage players in the game’s environment [21]. Three perspectives are evident in interactive narrative design—high-level abstract descriptions, dichotomic approaches, and descriptions of the design of specific pieces [10]. High-level descriptions take a holistic perspective to analyze problems from a distance. Although this perspective allows designers to see and consider the entire narrative, it fails to offer specific design advice for interactions in the narrative. The dichotomic approach focuses on framing a common platform particularly useful for multidisciplinary teams, however, as a result, forces ‘inclusion strategies’ instead of creating a combined design space. Finally, the third perspective describes specific design cases but can cause the design team to fail to identify generalizable conventions. In addition, the need for designers to be skillful in programming languages was emphasized for successful interactive narrative design [22]. To overcome these challenges the use of system narratives was suggested in which narrative and interaction are interrelated but notes that narrative designs should not entail preconceptions for a specific structure, but instead allow for complete freedom of the designer [10]. Although several design processes to outline and provide solutions to existing challenges are today known to facilitate interactive content narrative design, four phases were outlined [10]; (1) paper phase, (2) prototype phase, (3) production phase, and (4) testing phase. The first phase involves a generic goal or idea, which is further developed into a general outline of events to be visualized including the sequence of the narrative and character development. Typically, this phase concludes with a flow diagram or storyboard that integrates all aspects before developing a minimal viable product in phase 2. The prototype phase brings the storyboard to life and is a crucial step for the initial testing of the flow between the narrative and interaction. Final assets are adjusted in the production phase, which then moves to a group of beta users to test the design. For the purpose of our study, we test the effectiveness of interactive content narrative design by involving consumers in phase 1 and discussing the subsequent steps with immersive technology developers. We do this through co-creative design sprints that involve multiple consumers in comparable life stages to create narratives that are applicable and relevant to that specific target audience.

### 1.3. Interactive Narrative Design through Augmented Reality

The concept of narratives through the use of immersive technologies to trigger audiences’ cognitive and emotional processes has received much interest in the academic community, particularly in the area of marketing and retailing due to the capacity of narratives to engage consumers with the product and brand [9]. A prior study investigated the combination of storytelling through immersive technologies and suggested three distinct ways to effectively use storytelling through these technologies—reinforcing, reskinning, and remembering [23]. Reinforcing refers to the process of augmenting digital content to bring places to life, as multiple studies employed AR to bring showcase the state of heritage sites in the past [24]. The concept of reskinning is applied to reinterpret physical objects through augmented overlays. The use of mixed reality in this context is particularly interesting to give new meaning to existing objects in the physical environment in mixed reality narrative design. Finally, the process of remembering involves the retelling of stories in physical locations. For instance, the use of location-based AR videos was studied to bring personal stories of people in World War II back to life in areas around the Netherlands [25], not only to trigger emotional responses but also to give people a chance to share their personal stories of the past with others. A different study revealed that narrative storytelling through augmented reality influenced consumers’ positive attitudes toward the experience resulting in positive post-experience behavior [9]. However, we argue that triggering behavioral responses requires narratives to be emotionally stimulating and relatable to consumers’ personal contexts. Breaking established habits requires catching the attention of consumers during the decision-making process that can be repeated over time through conscious environment design choices [26]. In this light, it is imperative that the narrative design as well as interactive design matches the target audience in content relevance and capability of interaction. In our study, we thus use a participatory co-creative design process to involve consumers at the first stages of the interactive narrative design to develop impactful immersive sustainable food experiences through the employment of immersive technologies. 

## 2. Materials and Methods

### 2.1. Study Design

Three consumer co-creative design sessions were organized on two days on 20 and 21 April 2022 at a European institute followed by a focus group with mixed reality developers. The chronological order followed Knapp et al.’s five-step design spring process, understand, sketch, decide, prototype, and validate [27]. The first four steps were executed in the consumer co-creative design sessions, and the focus group was used to validate the outcomes with developers. 

**Figure 1:**
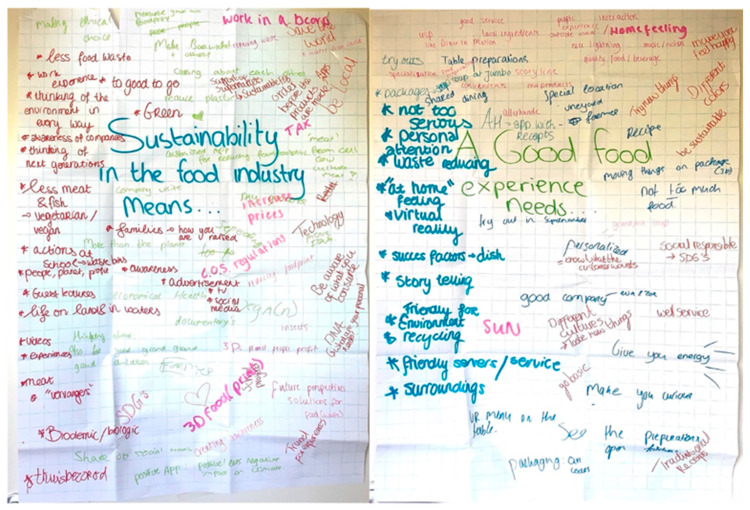
Illustration of Graffiti Walls.

For each step in the consumer co-creative design session, a different design tool was used and participants did not receive information on the goal of the research to avoid bias and framing. The session started with an introduction of the participants and the facilitator, an explanation of the design session, and the energizer as a pre-step of the process to boost creativity and help participants to be more alert and active [28]. The first step aimed to *understand* the context and challenge on which the narrative would be developed. We used ‘Graffiti Walls’, in which participants were invited to complete two open sentences on the topic of sustainability—*“Sustainability in the food industry means…” and “A good food experience needs…”* (Figure 1). 

After completing the first brainstorming step, ‘reframing’ was used to shift participant perspectives on the topic with the aim to inspire original and creative approaches to sustainability in the food context. Participants were asked about the positive or negative effects of sustainability and if they could be turned into food experience concepts. In the *Sketch* step, the group was divided into two subgroups to each generate their own narrative relevant to their personal lives using a storyboard with the aim of identifying what was important to them to be triggered as consumers to more sustainable food choices. The following guidelines were provided to facilitate the design of the narrative:

The story aims to trigger people like you to make more sustainable food choices.

The narrative entails a minimum of 10 scenes.

Focus on the dialogue and sequence in the narrative instead of visuals. 

The developed storyboards were presented to a focus group consisting of experts working in development teams for immersive technology applications and discussed for their originality and applicability to interactive content narratives using mixed reality. The focus group lasted approximately 90 min and started with a general discussion on interactive narrative content development through the use of immersive technologies before presenting two storyboards out of which one was selected for in-depth discussion. All sessions were documented with notes during the sessions and video recorded.

### 2.2. Participant Sample

A non-probability prefix consecutive sampling method was chosen for this study for consumers as well as developers. All participants had previous experience using Microsoft HoloLens 2 mixed reality glasses. They were screened for their interest in food and the food industry, foundational knowledge of sustainability issues within this context, and their ability to understand, speak and write in English. All participants were informed about the context of the study beforehand by e-mail, participation was voluntary and withdrawal from the study could be done at any time. In addition, a consent form was signed before the sessions to video record all sessions. In total, three consumer co-creative design sessions were conducted (N = 2, N = 4, N = 5) lasting an average of 2 hours each. In total, 4 male participants and 7 female participants who were all from generation Z participated in this study, who were mostly from the Netherlands with some internationals from India, Italy, Germany, and Taiwan. They were coded as PGn.N. to indicate the Group number (Gn) and Participant number (N). This was followed by an expert focus group (N = 4) to validate proposed consumer stories into AR solutions. The expert focus group consisted of three AR application developers with experience in immersive technology content design in particular in the realm of games and narrative design. 

### 2.3. Analysis and Evaluation

Reflexive journaling [29] was used to inductively evaluate the effectiveness of different stages of the design sessions and to determine whether the selected tools contributed to achieving the objective of the session. To determine if the consumer co-creative design sessions were effective, two tests were carried out [30]. Test 1 was the success assessment that analyzed the stories produced and determined the success potential of the stories. Test 2 was the application evaluation and examined how well the design sessions functioned. To evaluate the success potential of the developed stories in test 1, a focus group was conducted with immersive technology developers to assess how the narratives could be translated into an immersive mixed reality experience. Both the consumer design sessions, as well as the focus groups with developers, were analyzed using thematic analysis.

## 3. Results

### 3.1. Consumer Co-Creative Design Session Outcomes

**Figure 2:**
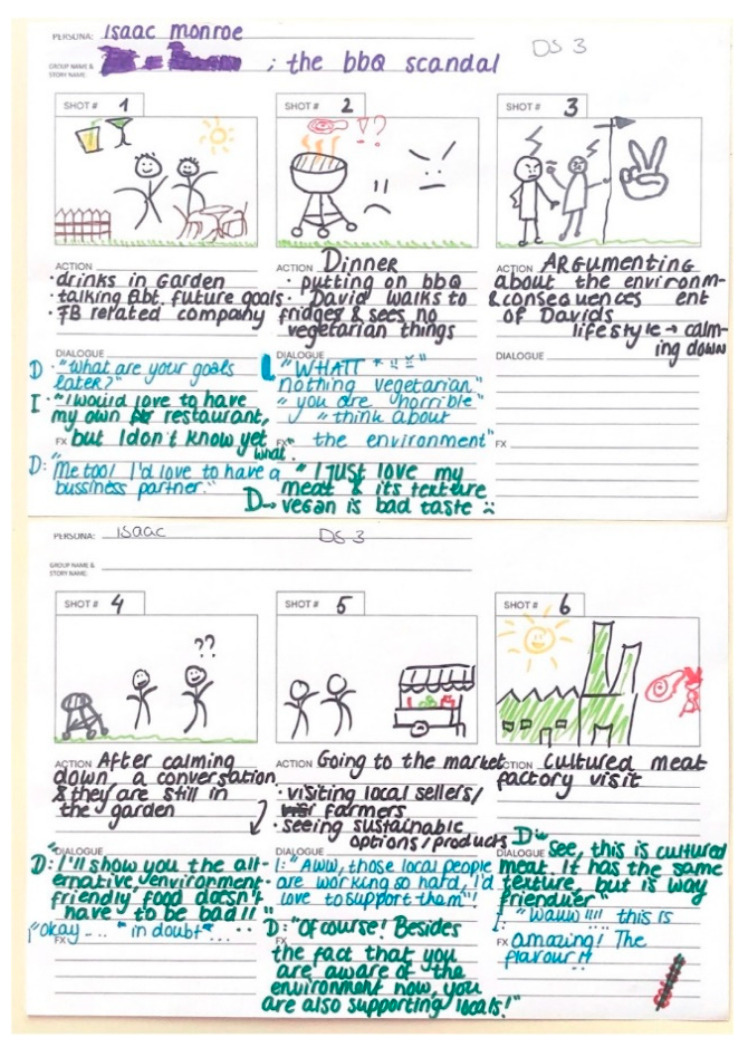
Illustration of the resulting consumer narrative using a storyboard.

Three themes emerged in analyzing the effectiveness of the developed narrative of all design sessions-(1) dramatic development of the narrative, (2) immersive narrative potential, and (3) a call to action toward sustainable approaches in food consumption. Storyboards were used to produce the narrative (see Figure 2 for example). 

#### 3.1.1. Dramatic Development of the Narrative

Participants realized that the development and inclusion of a dramatic arc in the narrative required a fully developing scenario in which a character could grow rather than a single scene. In design session 2, it was evident that the explicit communication of character emotions was key to developing a dramatic arc in a short narrative. This was further elaborated in design session 3, in which participants developed a narrative including multiple scenes and environments, allowing characters to be projected in multiple contexts, each eliciting its own set of emotions in characters. However, while this allowed for a sequential development of the narrative that was easy to follow, participants argued that it blurred the clear message of the narrative, which needed more emphasis due to the presentation of various scenes and character developments.

“Missing the call to action a bit. I think there is a good message, but it should be expressed better. By adding one extra scene after shot 9 to express even more emotions. Or adding a scene after shot 10 that explains why they chose the other restaurant.” (P3.2).

#### 3.1.2. Immersive Narrative Potential 

Discussing the developed stories among participants seemed to provide many insights for improvement that could result in refining narratives to be more impactful and relatable to the consumer. Participants emphasized that for narratives to speak to the audience, they needed to be relatable, indicating that aspects such as cultured meat were not well-known in the consumer market, and thus unrelatable to current consumers (P3.1: “*cultured meat is not really on the market yet, which makes this story a bit unrelatable. But it is nice for the future”.)* P1.1 added that for stories to be immersive to the audience they needed to be relatable not only in context but also in the involved characters, saying *“Emily (story protagonist) was frustrated in the entire story; this makes it hard to empathize and relate to her”.* P2.1 argued for the development of their narrative relating to supermarkets to share more information on available products; this was a feature that both participants were missing in their personal context. Thus, a narrative that outlines a desire in one’s personal context was regarded to have a higher immersion potential. In design session 3, a narrative involving a dialogue between two characters was presented in first-person view. It was argued that this change in perspective from a passive bystander who is following a narrative to an active participant in the narrative itself made it more relatable and engaging. P3.3 stated, “*I liked that they presented it as a conversation, like a role play. It is a conversation between two friends which makes it a more amusing story”.* Taking a first-person perspective in developing narratives in AR potentially offers seamless integration of the technology with interactive content narratives due to the current technological approach of AR devices such as headsets and AR smart glasses rather than hand-held devices. 

#### 3.1.3. Call to Action toward Sustainable Approaches in Food Consumption

In all three sessions, it was evident that participants were much more focused on the chronological development of the narrative. As a result of the Graffiti Wall preceding the storyboard, it was pointed out that influential narratives should include a clear message or call to action. This is particularly imperative for triggering thoughts and actions relating to sustainability practices among consumers. For instance, P1.4 noted, *“I am missing the sustainable call to action in this story. I understand it is about educating yourself, however, after listening to the story, it does not make me want to educate myself”.* In addition, narratives were considered to be more effective if the message of the narrative and potential call to action were framed as an empowerment of the consumer. Although Emily’s story included an outline of issues relating to restaurants and the food industry, it was seen as educational but ineffective to stimulate consumer behavior, as *“this story didn’t really move (participants) to make more sustainable choices”* (P1.2). Participants in design session 3 were equally skeptical, questioning “*do you think the impact of the local market will be enough to change Isaac’s perspective on food? Most of the time an experience will not totally change people their behavior”* (P3.2). Participants in design session 2 argued that a call to action could be further stimulated by triggering an emotional shock to a relatable character in the narrative. For instance, this could be achieved by informing consumers about the amount of CO_2_ emissions from eating meat. However, participants shared that it was imperative for consumers to be able to relate to provided numbers and statistics. This could be achieved through visual comparisons with familiar objects, such as comparing the number of emissions to the number of soccer fields. This approach could address a key challenge of consumers not being able to make sense of provided data and numbers on sustainability issues. 

#### 3.1.4. Evaluation of Design Sessions

All three design sessions underwent the same approach in evaluating the effectiveness of tools that were used during the sessions. Through the use of reflexive journaling, it became evident that the tools adopted in the design sessions reached their purpose, however, required to be applied through an interconnected approach. To allow for transparency and honest discussions among participants in the design of highly relatable narratives, it was crucial to establish a safe environment in which personal thoughts could be openly shared. A safe and comfortable environment for all participants is imperative, as relatable narratives require the involvement of personal aspects that participants can identify with. An ice-breaker exercise offers opportunities to establish a familiar basis in a short period of time. However, it was revealed that generating homogeneous groups for design sessions could further speed up this process, as people quickly grasped each other’s explanations and perspectives. This was particularly important for design session 2, which involved two participants that were unfamiliar with one another. Comparing the narratives and following discussions with the other design sessions, it can be concluded that a group that is familiar with one another seems to more openly engage in discussions and provide constructive feedback to each other. In this process, it was revealed that the objective and call to action of the narrative needed to be clear to everyone involved in the discussion to provide constructive feedback and areas for improving the effectiveness of the narrative. This could be further stimulated through the use of *Reframing* which was employed as a tool to make different associations to common topics (e.g., sustainability) to identify additional perspectives for the narrative design. P1.3 noted,

“Due to the reframing questions, you hear a lot of personal opinions and thoughts. It is interesting as it provoked a discussion. It helps to get a bit more in-depth on certain topics. For example, this group had different nationalities, it brought up some negative or positive experiences from their country. I grew up in India and Oman and had never barely heard of sustainability, let alone see some sustainable actions. However, when I got to the Netherlands it was everywhere around me.”

However, the design sessions exposed that the prompts used in the reframing process could have a strong influence on the focus and development of the narrative by consumers. Thus, prompts need to be posed with care and allow for interpretation among participants (e.g., *“How can you make a concept of this point?; How do you achieve this point?”*). Although Graffiti Walls were considered a useful exercise to brainstorm and frame the context of the topic at hand, it was evident that explicitly connecting the outcomes of the Graffiti Walls to the following narrative design helped consumers to specify a clear message and call to action to solve an identified issue at hand. P2.2 noted, *“when you started asking us those questions, you were able to send us in the right direction and help us get more detailed ideas”.* The design sessions revealed that using Graffiti Walls as a step to extract related personal values (e.g., awareness, curiosity) in the reframing process was a pragmatic approach to develop narratives that could become highly relevant and effective in their message. For instance, ‘awareness’ was noted down multiple times, while in the reframing process, aspects such as ‘actions at school’, ‘guest lectures’, and ‘awareness of consumption’ were added, offering more specific building blocks to develop a relatable content narrative. All participants concluded that presenting and discussing the designed narratives with each other assisted in the process of identifying which aspects were truly valued by consumers and which required more emphasis throughout the narrative to enhance the clarity of the message. 

### 3.2. Expert Focus Group Outcomes

The expert focus group lasted approximately 80 min and included a discussion with three experts of various backgrounds relevant to AR content development. Experts were prompted to discuss generic perspectives of effective content narrative design in AR before they were presented with two storyboards that were developed by consumer participants and invited to elaborate on their approach and the effectiveness of presenting these through AR. A total of four key themes were identified in the expert focus group which are outlined in this chapter.

#### 3.2.1. Instant Engagement through Interaction

A key aspect of effective content narrative design was argued to be interaction. Following the findings of earlier studies [31], interaction with content through technology helps consumers to become engaged and involved in the content. This is particularly true for AR, as interaction with digital content in the direct environment can create immediate engagement. However, experts discussed that it was vital to clearly understand the target audience and their habits of using technology to design interactive solutions and necessary instructions that are suitable and follows the logic of the audience.

*“E2: For me, I mean the main reason is that it’s the unique thing about this medium. If you don’t have interaction, you might as well make a movie, right? You want the person to be involved. And I think that’s also the reason why we have interaction because interaction creates buy-in almost immediately because you have to have to be involved.*

*E1: And you go into immersion again. But is also the curve of the story, right. It needs to be interesting enough to keep someone hooked from start to finish. There’s a lot of video games for example, you start and you know, “I’m not going to finish this one.”*

As this study aimed to maximize the impact on the attitude and behavior of consumers through interaction with content narratives, E1 mentioned that earlier designs focused on the repetition of interactions to build habitual traits which could be the key to overwriting old patterns or adapting new consumer behaviors. However, a key consideration was identified in this discussion that this approach was not well suited for narrative designs as there was a key difference in the context between a forced situation, where repetitive interaction can be manifested compared to situations where voluntary action through the internal motivation of consumers is desired.

*“E1: We had a facility that helps people with alcohol addiction. […] So they train it in virtual reality in a realistic environment to see if that helps. And then the trick is for them to make it as real as possible and have triggers. So we’ve put real models with their favorite brands. We put their favorite drinking locations in there, a beach, a forest, the park at home, whatever. […] And their trick was to repeat it a lot. So if you repeat it a couple of times in VR they get used to doing it. Then maybe in real life it gets easier.*

*E2: Yeah, but it doesn’t really work for narrative things like this. I think if you had the thing where you go to a supermarket and it highlights the differences between stuff, right then that would work because you keep doing it and you get used to what the differences are until you start noticing yourself.*

*E3: And in this specific case it was a therapeutical set. And in those settings, you are being forced to do it. And in this case, it needs to be a voluntary action that you take. So it needs to be something you actively do. And it’s only if you actively engage with the content.”*

#### 3.2.2. Understanding the End-User for Impactful Narratives

Following previous findings, to design relevant content narratives that speak to consumers, it was repeatedly mentioned that an in-depth understanding of the target audience should be one of the first steps in the design process. Thereby, the key question to be addressed should revolve around how to make consumers become invested in the narrative and its message. Within the context of game design, it was argued that providing character background stories and information was often a common approach to help consumers build emotional bonds with characters. However, external validation was also mentioned as a way to inspire consumers to be engaged with the narrative. For instance, current social media use patterns could be exploited to stimulate consumers to share their experiences and point of views of the designed narrative.

In this respect, it was suggested that a prompt and unfinished ending was often used in the games and entertainment context to stimulate consumers to think about or imagine the ending through their own interpretation, with the aim of enhancing the message and longevity of the narrative. AR offers promising avenues for future research in this respect to build true mixed realities through the blending of virtual content narratives with resulting consequences in the physical environment (e.g., selecting a food tasting in AR and having it delivered in real life). Experts agreed that this type of true mixed reality could maximize the impact of digital content consumption.

*“E3: For me, if you have an experience where people go through the experience and end up there, then from that point onwards you would leave it up to the people to actually start thinking like what you have with the little cards where the cards gives you the information, if you stop at that point, then you give people the room to think about something.”*

It was evident, however, that experts believed current consumer habits of interacting with AR to be limited and to rarely involve the consumption of narratives or entertainment. Instead, AR was argued to be limited to AR filters and overlay of factual information onto the physical environment. It was discussed that current cases of technological use and limitations as well as user acceptance and adoption of such technologies therefore needed to be considered for the design. Consequently, experts agreed that VR currently offered the more logical technological choice for designing narrative content. At the time of this research, potential workable alternatives could include a designated physical location where virtual content could be provided in a controlled setting in which consumers engage in the complete experience.

*E3: How I would see something like this being deployed right now is, say, a food-based experience center. […] You go in, you have the little booth or you have your hall where you, together with three other people, would all have a headsets and you would get this story being told to you. After the story is being told, then you go through the Information Center which is a more physical experience and after that you have this testing center where you do then either end up with the cultured meat in front of you that you can experience. Then you would go through a narrative experience where you don’t just have the narrative itself, the story, the MR story, but where you would then be further engaged in the world of cultured meats, where you would in the end experience it and where everything will come together. Whereas this spikes the interest to not just go through the rest of the information, but actively engage with the rest of the information. […] Turn it into an experience rather than a singular piece of content. Because then you can also make rooms that can facilitate the kind of stuff that you see here.”*

#### 3.2.3. Clarifying Objectives

All experts concurred that the first step in the design process was to research the context at hand and to conduct field research and desk research to understand the dynamics of the given context and problem. This could be an argument against the effectiveness of a consumer-driven approach, as an initial identification of objectives will be based on subjective participant perceptions instead of a more objective research approach to synthesize various perspectives.

*“E3: So for me, the first step is always topic research. Get further information, talk to people, people in this case often being the client about more in-depth information, retrieving information on what’s the actual topic that you’re trying to approach. What they already know, what’s already out there, and just gathering a whole bunch of information. So that you can use that entire bin of information to create a very clear narrative in your head and to then scope down and create a set with constructs of elements that you would like to highlight and that are necessary within this project.*

*E2: I think what we do after we get kind of a briefing is to figure out how what approaches are there to solve this. […] I think it usually works best if you try to come up with semi-concrete ideas that you can pitch to the clients and then they should be able to highlight what parts they like and don’t like.”*

In contrast, it was also pointed out that developers often had a bias because of previous projects and work that would suggest possible directions for future projects. To overcome this bias, it was suggested that directly and indirectly involved stakeholders should be included in the initial identification of the design objective. Experts argued that it was crucial to have multiple perspectives on the context at the table for a thorough understanding of the context which could offer different ways of thinking about the issue at hand. Although this study made an attempt to prompt consumers to elicit a key objective or message in their narrative, experts suggested that in general, three key questions should be addressed in this process; (1) who is the target audience?; (2) what is the problem?; and (3) where do we want to go?

*“E3: There’s always a narrative within the experience that you create. And for specifically what we are building, the first thing that I would consider is what’s the goal of the project. For me, I always want to have a very clear visual insight, before I know what the interactions are going to be or what level of interaction is necessary. I’m specifically not thinking about MR in this case. For me it will be ‘what’s the goal? Where do we want to end up or what do we want someone to take away or learn from this experience?’*

*E1: It often comes from a problem.*

*E2: And of course, with that person. That’s where it starts.*

*E3: Yes. Like, who do we have? What’s the problem and where do we want to do?”*

Designing character backgrounds that consumers did not identify with could greatly limit the perceived impact of that character on consumers. Having characters representing the key objective of the narrative that consumers did not agree with could potentially put the clarity and effect of the objective in jeopardy. In games and entertainment characters were often projected to elicit traits that the audience could identify with and build an emotional bond to. As such, to reach a high-impact objective, it was crucial that consumers could establish a bond with the narrative or characters within.

*“E2: So that that’s one big thing I would change here.*

*E1: Yeah, you run the risk that if your viewer doesn’t like Isaac. […] Maybe a bit risky there.*

*E3: It can be an immediate annoyance. There’s no build up. So you don’t get invested into the story before something so impactful happens that in that moment you decide, “I like it or I do not like it.” And if you do not like it, you just immediately switch off. You will not continue anymore.”*

#### 3.2.4. Pre- vs. Post-Consideration of Narrative Medium

Views on whether the medium to present the narrative should be considered in the initial phase or later were divided. On the one hand, experts argued that it was generally preferred not to think too early about the technology itself, as it created the risk of limiting potential approaches to how the technology was used as well as designing appropriate content narratives. In light of this, budget allocations per project were discussed as a way to narrow down a potential list of solutions. However, on the other hand, it was argued that consideration of technology in the initial phases could take technological possibilities into consideration when designing the narrative, creating a logical fit between these two aspects. This issue was illustrated in discussing the narratives designed by participants.

*“E2: First thing, that’s a problematic approach, because now you’re taking something that’s kind of meant for just a video, right? I think that’s what was in mind when making this. And you’re saying, “How do we use it in MR?” You could do that, but you probably won’t make optimal use of what MR could offer? Because you designed it for a different medium. […] Because this seems like a linear narrative, right? So what kind of interaction can you put in there? You can make a choice in there. You can make some of those small things that you interact with to go to the next step basically, but those are not very interesting most of the time.”*

According to E3, creating the narrative without consideration of the medium could limit the potential that the technology could offer. For instance, AR could offer an opportunity to involve consumers more in the narrative from a first-person perspective. It was argued that a dedicated user-perspective dictates which technology would be more suitable to use as the medium. For the designed participant stories, which were narrated from a third-person perspective, VR was therefore argued to be more suitable as the narratives were following a character instead of directly involving the user.

*E3: This specific example could lend itself better for a low interaction narrative experience where the idea is not that you actively participate in a story, but that you’re able to, in a more immersive way view the content that’s being given. Because in the end the whole thing about MR is that you are given a more ‘on-site’ approach to experiencing a narrative. And you can put something on the table, but for me that doesn’t add a lot in this specific case. So with this I would rather go VR than AR. I think that makes way more sense in this specific case. Because in this case, it’s about experiencing this specific narrative. And if you want to experience that specific narrative, as in being there, then virtual reality is just a better medium.”*

## 4. Discussion

### 4.1. Evaluation of Consumer Co-Creative Narrative Designs

Consumer-driven narrative design appears to be a valuable approach as part of the initial user research to identify important elements that speak to the consumer. In consideration of the entire design process, this step can allow for the pursuit of a diverging approach before focusing on key elements that were perceived as important for consumers to converge into specific design alternatives. However, experts warned against clients designing the content, as it could result in opposition to potential future changes which could foster difficulties in producing the best possible solution due to the client being too invested in what they had already produced. Instead, effective consumer co-creative narrative design methodology needs to allow consumers to design narratives with sufficient freedom of interpretation without providing too much direction, such as establishing a character in full or providing the scope of a context. Design tools such as Graffiti Walls are suitable for providing freedom of interpretation and associations with topics. To allow for multiple interpretations to emerge, an alternative approach could be to prompt consumers to write one narrative about themselves as the lead character, and one about ‘others’ as the lead character to obtain input on who they perceive to be opposite to them. 

The narratives designed by participants in this study resulted in largely straightforward messages that were perceived to be too stereotypical and predictable. The adopted approach in this study entails the difficulty of working with consumers who are not professional storytellers, and thus are not aware of storytelling techniques that could benefit the flow and immersion of an effective narrative. As a result, there is a risk of creating narratives that are not highly attractive to general consumers. Developing a more subtle message in the narrative could offer space for interpretation which could increase the impact of the message. 

Continuous consumer testing was identified as a crucial aspect of the design process. Testing could be critical to revealing, early in the process, when the consumer might not agree with the solution. This allows for early identification of issues in the design that might have been overlooked or wrongly interpreted. However, it needs to be acknowledged that iterative feedback on narrative design has been a challenge, as consumers generally do not know what they liked or disliked in evolving narratives compared to product features. 

### 4.2. Proposed Framework for Effective Participatory Co-Creative Narrative Design

Based on the results of our consumer study, we see indications that Knapp et al.’s traditionally linear intended design approach could be improved by introducing back loop feedback moments into the methodology.

**Figure 3:**
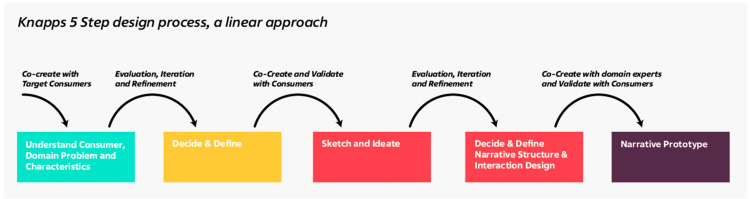
5-step design process according to Knapp et al.

Looking at the design process as a whole, iteration and validation are primarily focused on the results of the individual stages of the narrative creation process (see Figure 3). In our study, we adopted a peer-to-peer feedback moment to reflect and improve on the designed narratives. In the case of designing narratives for AR, it is essential to note that the media context changes fundamentally between the individual steps, whereby the results of each stage influence the following ones. Consequently, an initially evaluated result (e.g., the results from the Graffiti Wall) might behave differently in another context (e.g., multimodal interaction in AR). 

**Figure 4:**
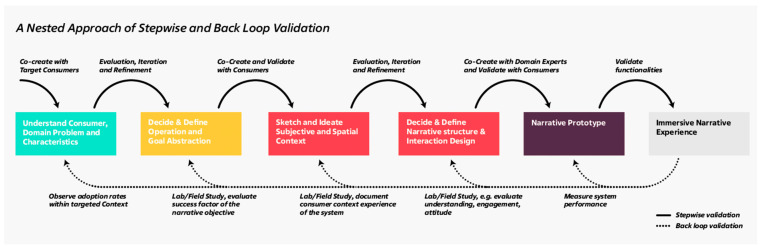
Framework for consumer participatory co-creative narrative design for immersive narrative experiences: A nested approach of stepwise and back loop validation cycles.

Consequently, we propose to describe the design process as an iterative but nested collection of hypotheses that lead to the final narrative prototype (Figure 4). A nested approach addresses several critical shortcomings that are evident in current design processes. For instance, even if the resulting narrative works efficiently in the intended context, we cannot conclude that every individual stage led to optimal results. Similarly, if the system fails, the individual stages, like the initial domain context definition, do not necessarily need to reflect the correct perspectives. A general asymmetry of knowledge and experience between the relevant stakeholders (consumers, developers, narrative design experts) exists, especially at the sketch, decide, and prototype stages. With this in mind, a careful justification is needed to understand individual stages within the design process and their implications within the resulting narrative.

Within the stepwise validation process, the respective results of the individual stages are validated and condensed toward the intended narrative context, primarily together with consumers. However, a significant shortcoming here is that the validation is limited to the individual design step, and the transferability of the results is not guaranteed. Therefore, the back loop validation focuses on the validation of the narrative artifact with regard to the individual stages. Back loop validation can only be conducted if a narrative artifact exists. The first step is aiming at the application’s technical applicability and evaluating the system’s technical performance (e.g., memory usage, framerate stability). An unstable system would influence every following stage of the back loop cycle. If the system works, the cognitive and non-cognitive effects are related to the content of the narrative (e.g., understanding, engagement, attitude). In the next step, the evaluation focuses on the user’s reaction to the expression of the narrative artifact (e.g., user experience). After that, it needs to be carefully justified if the narrative achieves the initially defined goal and finally observe if the developed solution is adapted and leads to a measurable change in sustainable food consumption, indicating the overall efficiency.

## 5. Conclusions and Future Research

This study aimed to evaluate the suitability of a design process to test its effectiveness for consumer participatory co-creative narrative design for immersive sustainable food experiences in augmented reality. The results of the study stressed several points for adjustment of a conventional design process which was revealed to be limited in their back loop validation capabilities of specific design steps. In particular, the findings of this study suggest that consumer co-creative design processes that aim for impactful narratives need to allow for sufficient freedom of interpretation and meaning creation within the initial stages. Various design tools can be supportive of this diverging phase (e.g., Graffiti Walls) until a clear objective for a narrative design is formulated. A crucial aspect of technology-mediated consumer-designed narratives, such as in the case of interactive content narratives in AR, is that media-context can shift drastically while moving through the design process. At the time of this study, AR still holds limited use cases in the consumer market, making it challenging for consumers to consider the full capacity of the technology in their narrative design. This can have profound implications on the level of impact of designed and presented narratives, potentially reducing their effectiveness for stimulating sustainable consumer attitudes and behavior. To address this challenge, we propose a framework for consumer participatory co-creative narrative design that considers several back loop validation steps to evaluate the effectiveness of each step in the design process (Figure 4). We posit that a systematic approach to validation in interactive narrative design is needed to be able to analyze individual components in the design and consider efficient content and system-specific iterations to create impactful immersive narrative experiences for consumers. The involvement of target consumers early in the design process is crucial to scope a context and narrative that is highly relevant and appealing and that speaks to the audience. However, further research is needed to identify possible methodologies across the validation process and to test the suitability of methods in various contexts. For instance, designing interactive content narratives in AR requires careful consideration of embedding technological capabilities to logically fit the narrative. As such, it greatly benefits from the co-creative sessions with consumers and developers involved with the respective technology to identify the purpose of narrative designs in relation to fitting technological solutions in the design process. Furthermore, we advocate for future research to identify key success factors in the back loop validation process to determine the effectiveness of specific design steps in their ability to evoke sustainable attitudes and behavior in consumers. Determining success factors that are measurable and comparable across contexts is expected to provide us with a common benchmark to evaluate the effectiveness of immersive narrative designs in this field. 

## Data Availability

All data of this study were handled anonymously and in full compliance with the GDPR and the Netherlands Research Integrity Code. The data is stored for the long term on Microsoft Sharepoint of Breda University of Applied Sciences for further processing and use. Microsoft Sharepoint of Breda University of Applied Sciences is a safe and secure environment supported by the IM/ICT department of BUas.

## References

[1] Jaca C., Prieto-Sandoval V., Psomas E.L., Ormazabal M. (2018). What should consumer organizations do to drive environmental sustainability?. J. Clean. Prod..

[2] Griskevicius V., Cantú S.M., van Vugt M. (2012). The Evolutionary Bases for Sustainable Behavior: Implications for Marketing, Policy, and Social Entrepreneurship. J. Public Policy Mark..

[3] World Health Organization (2011). Global Status Report on Noncommunicable Diseases 2010. who.int/nmh/publications/ncd_report_full_en.pdf.

[4] Pfeiffer J., Fegert J., Greif-Winzrieth A., Hoffmann G., Peukert C. (2021). Can Immersive Systems Help Address Sustainability Goals? Insights from Research in Information Systems. Market Engineering.

[5] Bailey J.O., Bailenson J.N., Flora J., Armel K.C., Voelker D., Reeves B. (2015). The Impact of Vivid Messages on Reducing Energy Consumption Related to Hot Water Use. Environ. Behav..

[6] Fox J., McKnight J., Sun Y., Maung D., Crawfis R. (2020). Using a serious game to communicate risk and minimize psychological distance regarding environmental pollution. Telemat. Inform..

[7] Shin D. (2018). Empathy and embodied experience in virtual environment: To what extent can virtual reality stimulate empathy and embodied experience?. Comput. Hum. Behav..

[8] QReal (2022). Augmented Reality Food. Use Cases. https://www.qreal.io/use-cases/augmented-reality-food-use-cases.

[9] Sung E., Han D.-I.D., Bae S., Kwon O. (2022). What drives technology-enhanced storytelling immersion? The role of digital humans. Comput. Hum. Behav..

[10] Koenitz H. (2015). Design Approaches for Interactive Digital Narrative. Proceedings of the International Conference on Interactive Digital Storytelling.

[11] Holmlid S., Mattelmäki T., Visser F.S., Vaajakallio K. (2015). Co-Creative Practices in Service Innovation. The Handbook of Service Innovation.

[12] Galvagno M., Dalli D. (2014). Theory of value co-creation: A systematic literature review. Manag. Serv. Qual. Int. J..

[13] Marlien R.A., Alimuddin R.R., Priyanto S.H., Andadari R.K., Ihalauw J.J. (2019). Co-synergy and Co-creation Value on Customer Behavioural Outcomes. Proceedings of the International Conference on Banking, Accounting, Management, and Economics (ICOBAME 2018).

[14] Antón C., Camarero C., Garrido M.-J. (2018). Exploring the experience value of museum visitors as a co-creation process. Curr. Issues Tour..

[15] Kazadi K., Lievens A., Mahr D. (2016). Stakeholder co-creation during the innovation process: Identifying capabilities for knowledge creation among multiple stakeholders. J. Bus. Res..

[16] Lang K., Shang R., Vragov R., Incentive L.T.R., Baruch College, Long Island University Brooklyn, Mount Saint Mary College (2015). Consumer Co-creation of Digital Culture Products: Business Threat or New Opportunity?. J. Assoc. Inf. Syst..

[17] Ramaswamy V., Ozcan K. (2014). The Co-Creation Paradigm.

[18] OHern M.S., Rindfleisch A. (2010). Customer Co-Creation: A typology and research agenda. Review of Marketing Research.

[19] Bizzocchi J., Woodbury R.F. (2003). A Case Study in the Design of Interactive Narrative: The Subversion of the Interface. Simul. Gaming.

[20] Murray J.H. (1997). Hamlet on the Holodeck: The Future of Narrative in Cyberspace.

[21] Strohecker C. (1997). A Case Study in Interactive Narrative Design. Proceedings of the 2nd Conference on Designing Interactive Systems Processes, Practices, Methods, and Techniques.

[22] Spierling U., Szilas N. (2009). Authoring Issues Beyond Tools. Interactive Storytelling.

[23] Azuma R., Barfield W. (2015). Location-Based Mixed and Augmented Reality Storytelling. Fundamentals of Wearable Computers and Augmented Reality.

[24] Han D.I., Jung T., Gibson A. (2013). Dublin AR: Implementing Augmented Reality in Tourism. Information and Communication Technologies in Tourism 2014.

[25] Calvi L., Hover M. (2021). Storytelling for Mythmaking in Tourist Destinations. Leis. Sci..

[26] Pinder C., Vermeulen J., Cowan B.R., Beale R. (2018). Digital Behaviour Change Interventions to Break and Form Habits. ACM Trans. Comput. Interact..

[27] Knapp J., Zeratsky J., Kowitz B. (2016). Sprint: How to Solve Big Problems and Test New Ideas in Just Five Days.

[28] Institute of Development Studies (2022). Energizer|Participatory Methods. Institute of Development Studies. https://www.participatorymethods.org/glossary/energizer.

[29] Lincoln Y.S., Guba E.G. (1982). Establishing Dependability and Confirmability in Naturalistic Inquiry Through an Audit.

[30] Blessing L., Chakrabarti A. (2009). DRM, A Design Research Methodology.

[31] Sung E., Bae S., Han D.-I.D., Kwon O. (2021). Consumer engagement via interactive artificial intelligence and mixed reality. Int. J. Inf. Manag..

